# Kidney Dysfunction and Its Progression in Patients Hospitalized Duo to COVID-19: Contribution to the Clinical Course and Outcomes

**DOI:** 10.3390/jcm10235522

**Published:** 2021-11-25

**Authors:** Katarzyna Kilis-Pstrusinska, Katarzyna Akutko, Joanna Braksator, Anna Dancewicz, Patrycja Grosman-Dziewiszek, Tatiana Jamer, Katarzyna Juszczyńska, Klaudia Konikowska, Marta Koruba, Małgorzata Pupek, Agnieszka Rusiecka, Krzysztof Kujawa, Barbara Adamik, Adrian Doroszko, Krzysztof Kaliszewski, Agnieszka Matera-Witkiewicz, Michał Pomorski, Marcin Protasiewicz, Janusz Sokołowski, Katarzyna Madziarska, Ewa A. Jankowska

**Affiliations:** 1 Clinical Department of Paediatric Nephrology, Wroclaw Medical University, Borowska Street 213, 50-556 Wroclaw, Poland; marta.salamacha@gmail.com; 2 2nd Clinical Department of Paediatrics, Gastroenterology and Nutrition, Wroclaw Medical University, M. Curie-Sklodowskiej Street 50-52, 50-369 Wroclaw, Poland; katarzyna.akutko@umw.edu.pl (K.A.); joanna.braksator@umw.edu.pl (J.B.); anna.dancewicz@umw.edu.pl (A.D.); tatiana.jamer@umw.edu.pl (T.J.); 3 Department of Pharmacology, Wroclaw Medical University, J. Mikulicza-Radeckiego Street 2, 50-345 Wroclaw, Poland; patrycja.grosman-dziewiszek@umw.edu.pl; 4 Department of Pharmaceutical Biochemistry, Wroclaw Medical University, Borowska Street 211A, 50-556 Wroclaw, Poland; katarzyna.juszczynska@umw.edu.pl; 5 Department of Dietetics, Wroclaw Medical University, Parkowa Street 34, 51-616 Wroclaw, Poland; klaudia.konikowska@umw.edu.pl; 6 Department of Biochemistry and Immunochemistry, Division of Chemistry and Immunochemistry, Wroclaw Medical University, M. Sklodowskiej-Curie Street 48/50, 50-369 Wroclaw, Poland; malgorzata.pupek@umed.wroc.pl; 7 Statistical Analysis Centre, Wroclaw Medical University, K. Marcinkowski Street 2-6, 50-368 Wroclaw, Poland; agnieszka.rusiecka@umw.edu.pl (A.R.); krzysztof.kujawa@umw.edu.pl (K.K.); 8 Clinical Department of Anaesthesiology and Intensive Therapy, Wroclaw Medical University, Borowska Street 213, 50-556 Wroclaw, Poland; barbara.adamik@umw.edu.pl; 9 Clinical Department of Internal Medicine, Hypertension and Clinical Oncology, Wroclaw Medical University, Borowska 213, 50-556 Wroclaw, Poland; adrian.doroszko@umw.edu.pl; 10 Clinical Department of General, Minimally Invasive and Endocrine Surgery, Wroclaw Medical University, Borowska Street 213, 50-556 Wroclaw, Poland; krzysztof.kaliszewski@umw.edu.pl; 11 Screening Laboratory of Biological Activity Tests and Collection of Biological Material, Wroclaw Medical University, Borowska Street 211A, 50-556 Wroclaw, Poland; agnieszka.matera-witkiewicz@umw.edu.pl; 12 Clinical Department of Gynecology and Obstetrics, Wroclaw Medical University, Borowska Street 213, 50-556 Wroclaw, Poland; michal.pomorski@umw.edu.pl; 13 Clinical Department and Clinic of Cardiology, Wroclaw Medical University, Borowska Street 213, 50-556 Wroclaw, Poland; marcin.protasiewicz@umw.edu.pl; 14 Department of Emergency Medicine, Wroclaw Medical University, Borowska Street 213, 50-556 Wroclaw, Poland; janusz.sokolowski@umw.edu.pl; 15 Clinical Department of Nephrology and Transplantation Medicine, Wroclaw Medical University, Borowska Street 213, 50-556 Wroclaw, Poland; katarzyna.madziarska@umw.edu.pl; 16 Centre for Heart Diseases, Wroclaw Medical University, Borowska Street 213, 50-556 Wroclaw, Poland; ewa.jankowska@umw.edu.pl

**Keywords:** COVID-19, kidney dysfunction, acute kidney injury, hospitalization, mortality

## Abstract

The disease caused by coronavirus SARS-CoV-2 (COVID-19) can affect almost all organs of the human body, including kidneys. We conducted a one-center study to comprehensively analyze the effects of kidney involvement on the course and outcomes in patients hospitalized with COVID-19, depending on the estimated glomerular filtration rate (eGFR) at admission. Out of the 1958 patients, 1342 (68.54%) had eGFR ≥ 60 mL/min/1.73 m^2^ (group A) and 616 (31.46%) had eGFR < 60 mL/min/1.73 m^2^ (group B). Group B was additionally divided into subgroups B1, B2, and B3 based on eGFR. We found that mortality rates during hospitalization, as well as after 90 and 180 days, were much higher in group B than group A. The highest mortality was observed in the B2 subgroup with eGFR of 15–29. The mortality of B patients was associated with comorbidities, respiratory dysfunction, immunological impairment, and more frequent development of AKI. AKI had a negative impact on patients’ survival, regardless of the initial renal function. At discharge, 7.4% of patients had serum creatinine levels 30% higher, or more, as compared to admission. The disease course and outcomes in COVID-19 patients are associated with baseline eGFR; however, AKI during hospitalization is a more significant predictor of poor prognosis regardless of the initial renal function.

## 1. Introduction

The disease caused by coronavirus SARS-CoV-2 (COVID-19) can affect almost all organs of the human body [1,2], including kidneys. The mechanism of kidney involvement in COVID-19 appears to be multifactorial. Thus far, data suggest effects of direct viral infection (viral tropism to the renal system), hypoxia, inflammatory syndrome-mediated injury, hemodynamic instability, vascular injury, and hypercoagulable state [3,4,5,6].

The number of papers concerning kidney involvement in COVID-19 is constantly growing. Most of them describe acute kidney injury (AKI) in patients without previous renal impairment [7,8,9,10]. Data concerning the prevalence of AKI in hospitalized patients with COVID-19 vary from 0.5% to 46% [4,9,11]. In a large multicenter study, AKI was reported in 36.6% of 5449 adult patients [10]. Chan et al. observed AKI in 68% of all patients admitted to an Intensive Care Unit (ICU) because of COVID-19 and 20% of them needed renal replacement therapy (RRT) [9]. Some data suggest a gradual reduction in the frequency of AKI in COVID-19, probably attributed to the rising proportion of younger patients with fewer comorbidities [4,12]. Existing data are heterogeneous because of patients’ race, ethnicity, age, population size, the severity of illness, time of analysis, and definitions of AKI. It is well known that AKI is associated with worse outcomes regardless of its etiology [13,14,15]. In the case of the current COVID-19 pandemic, the available data indicate a strong association between AKI and mortality [16,17,18]. Hirsch et al. reported mortality of 35% for patients with AKI compared to 6% in those without AKI [10].

There are definitely far fewer reports about the course of COVID-19 in patients with kidney dysfunction at admission to hospital, including chronic kidney disease (CKD) [19,20,21]. CKD is a global public health problem [22,23]. CKD can influence the course and outcome of a viral disease [24]. In a meta-analysis of 1389 COVID-19 patients, the odds ratio of occurrence of severe COVID-19 in patients with previous CKD reached 3 [20]. On the other hand, disturbed kidney function can be further diminished due to active viral infection, and people with kidney impairment may develop AKI progressing to renal failure [7,25]. However, the impact of COVID-19 on patients with initial kidney impairment has not been well defined so far. Different indexes are used for kidney function assessment but the estimated glomerular filtration rate (eGFR) remains one of the best markers [26].

Therefore, we conducted a retrospective one-center study to analyze the effects of kidney involvement on the course of disease and outcomes in patients hospitalized with COVID-19. The detailed study scheme involved a comparison of demographic and clinical characteristics, disease course, and outcomes in patients with eGFR ≥ 60 mL/min/1.73 m^2^ and eGFR < 60 mL/min/1.73 m^2^ as diagnosed at admission to hospital because of SARS-CoV-2. We also examined incidence and risk factors associated with AKI in both mentioned groups and the impact of these variables on outcomes. The findings may add to the evolving knowledge about the association between kidney function and COVID-19 and they may be useful in the management of patients with COVID-19.

## 2. Materials and Methods

### 2.1. Study Design and Participants

We retrospectively analyzed medical records of patients hospitalized at one medical center because of COVID-19 between February 2020 and June 2021.

The study protocol was approved by the Institutional Review Board and Ethics Committee of Wroclaw Medical University, Wroclaw, Poland (No: KB-444/2021). The routine data were collected retrospectively; therefore, written informed consent to participate in the study was not required. The Bioethics Committee approved the publication of anonymized data.

All patients were admitted to the hospital because of COVID-19 symptoms and tested positive for COVID-19. The testing was based on the protocol published by the World Health Organization (WHO) [27]. Nasopharyngeal swab specimens were taken in all patients and SARS-CoV-2 RNA was detected in the samples by reverse-transcription polymerase chain reaction (RT-PCR).

The follow-up period started on the day of hospitalization and ended the day of discharge or death. It was recorded for the entire duration of hospitalization. Further information about the patients’ deaths was collected after 90 and 180 days from admission. Patient characteristics were obtained from individual clinical records. Admission data included demographic information and clinical characteristics: Vaccination against SARS-CoV-2, breathing support, smoking, comorbidities, home medication, and laboratory results. In the present study, lab results at admission (blood count, C—reactive protein (CRP), serum procalcitonin (PCT) and interleukin-6 (IL-6), serum protein, albumin, creatinine, urea, uric acid, sodium, potassium, D-dimers, fibrinogen, APTT, and INR) and serum creatinine at discharge were analyzed. Parameters analyzed during hospitalization included the occurrence of pneumonia, intravenous application of loop diuretics, vasopressors and antibiotics, deterioration of the patient’s condition (any need for escalated oxygen therapy), patient transfer to ICU, and application of the most aggressive forms of breathing support. The measured outcomes were in-hospital mortality, 3-month and 6-month mortality after admission to hospital, and the end of hospitalization other than due to death (discharged to go home/emergency transferred to another center—deterioration/transferred to another center for rehabilitation).

All patients were first divided into two groups according to eGFR at admission to the hospital: Group A with eGFR ≥ 60 mL/min/1.73 m^2^ and group B with eGFR < 60 mL/min/1.73 m^2^. eGFR was calculated based on the Modification of Diet in Renal Disease [MDRD] Study Equation [26]. Patients were included in group A when they had negative prior medical history concerning CKD and/or prior to hospital admission their eGFR was ≥60 m/min/1.73 m^2^. They served as our reference group. Patients qualified for group B when their eGFR at admission was <60 mL/min/1.73 m^2^ and they were diagnosed as having CKD in the past or they had any chronic disease strictly connected with CKD, such as diabetes mellitus and hypertension. In addition, patients with eGFR < 60 mL/min/1.73 m^2^ were divided into four subgroups depending on the level of eGFR: Group B1 with eGFR of 30–59 mL/min/1.73 m^2^, group B2 with eGFR of 15–29 mL/min/1.73 m^2^, group B3a with eGFR < 15 mL/min/1.73 m^2^ without RRT, and group B3b with eGFR < 15 mL/min/1.73 m^2^ on hemodialysis.

The Disease Improving Global Outcomes (KDIGO) classification according to serum creatinine (SCr) criteria was used to define AKI’ i.e., an increase in SCr ≥ 1.5 times higher from the baseline value within seven days [25,28]. The criteria for AKI based on urine testing during hospitalization were not taken into consideration because of a lack of reliable data. In groups A and B, patients with AKI during hospitalization (regardless of the time of its onset) were recorded as AKI and patients without AKI as non-AKI. Patients with a clear diagnosis of AKI at admission were excluded from the study population.

### 2.2. Statistical Analysis

Descriptive data were presented as numbers and percentages for categorical variables, and as the mean, standard deviation, median, and interquartile range (IQ) for numerical variables. The distribution of continuous variables was tested using visual (histogram) and analytical methods (Kolmogorov–Smirnov/Shapiro–Wilk tests). The Chi-square test or Fisher exact test were used for the comparison of qualitative variables. The Mann–Whitney U test was used for subgroup analysis of non-normally distributed variables and Student’s *t*-test was used for the comparison of means for normally distributed data. In multiple group comparisons of numerical variables, the analysis of variance (ANOVA) test was used for normally distributed numerical variables and the Kruskal–Wallis test was used for non-normally distributed numerical variables. Kaplan–Meier analysis with the log-rank test was used to compare the survival experience of the patients to the 90th day from hospital admission, divided in the four groups based on eGFR at admission to the hospital (eGFR ≥ 60 m/min/1.73 m^2^ and eGFR > 60 m/min/1.73 m^2^) and those who developed AKI or not during hospitalization. The dependency of AKI appearing in COVID-19 patients on sets of predictors was assessed with the use of multivariable logistic regression. The best model subsets were selected with the use of the corrected Akaike Information Criterion (AICc). The models, for which the difference in AICc between the best model and their AICc values was below 2, were averaged using Akaike weights. The analysis was performed with the use of R package MuMIn [29]. All the statistical tests were two tailed and the statistical significance level was set at *p* < 0.05. The analyses were performed using Statistica v.13.3 (TIBCO Software Inc., Palo Alto, CA, USA), except multiple logistic regression, which was performed with the use of the R (open source software v. 4.0.4, Auckland, New Zealand) package MuMIn (v. 1.43.17) [29].

## 3. Results

The study group consisted of 1958 COVID-19 patients. There were 937 women (47.85%) and 1021 men (52.15%), and the mean age was 62.34 ± 17.57 years. Out of the 1958 COVID-19 patients, 1342 (68.54%) had eGFR ≥ 60 mL/min/1.73 m^2^ (group A) and 616 (31.46%) had eGFR < 60 mL/min/1.73 m^2^ (group B) at admission. Groups B1, B2, B3a, and B3b consisted of 409 (66.4%), 122 (19.8%), 43 (6.98%), and 42 (6.82%) patients, respectively.

### 3.1. Characteristics of Patients at Hospital Admission

Upon hospital admission, group A patients (with higher eGFR) differed from patients in group B (lower eGFR) by age, but also by their need for respiratory support, comorbidities, as well as the frequency of application of antihypertensive drugs, statins, and diuretics. Patients with eGFR ≥ 60 mL/min/1.73 m^2^ were younger. The percentage of patients without oxygen therapy was similar for both groups (54.81% in group A vs. 53.17% in group B), while among patients who needed oxygen application, advanced forms of oxygen therapy (HFNC, BiPAP/CPAP, respirator) were applied in a higher share of patients in group B as compared to group A. As far as comorbidities are concerned, the incidences of the following was significantly higher in group B than in group A: Diabetes (40.81% and 18.42%, respectively), hypertension (68.83% and 42.18%, respectively), other cardiovascular disorders (25% and 7.23%, respectively), and chronic obstructive pulmonary disease (COPD) (6.17% and 2.68%, respectively). Patients in group B were more frequently on antihypertensive drugs, statins, and diuretics. Table 1 shows the baseline demographic and clinical characteristics of the study participants.

In the case of laboratory parameters, differences between groups A and B concerned almost all items except for sodium and fibrinogen (Table 2). The group with lower eGFR recorded markedly higher values than the group with higher eGFR for CRP (67.89 mg/dL vs. 44.23 mg/dL, respectively), PCT (0.27 pg/mL vs. 0.06 pg/mL, respectively), and IL-6 (24.7 pg/mL vs. 15.3 pg/mL).

Within the group with eGFR < 60 mL/min/1.73 m^2^ (group B), subgroups classified by the degree of eGFR reduction at admission (B1-B3) did not differ by the type of initial respiratory support or incidence of hypertension. However, there were differences in the number of people with other cardiovascular disorders, applied antihypertensive drugs and diuretics, as well as age (elder patients in the B1 and B2 groups) (Table 3). B subgroups also differed in lab parameters, not only those reflecting the degree of kidney dysfunction (creatinine, urea, uric acid, potassium), but also blood count, CRP, and PCT. The details of lab parameters at admission by subgroups of B are presented in Table 4.

### 3.2. Course of COVID-19 vs. eGFR

A more severe course of COVID-19 was observed in patients in group B as compared to group A (Table 5). They were diagnosed with pneumonia more frequently (57.95% vs. 51.04%). Patients in group B required more intensive forms of oxygen support, more frequently they needed antibiotics, catecholamines, and intravenous loop diuretics. Deterioration of health during hospitalization was observed in 23.25% patients in group A and 39.12% in group B. Moreover, 13.47% patients of group B were transferred to ICU as compared to 9.54% of patients in group A.

In the group of patients with eGFR < 60 mL/min/1.73 m^2^, differences were found in the incidence of pneumonia, depending on the level of eGFR (61%, 55%, 40%, and 55% in groups B1, B2, B3a, and 3b, respectively). However, those patients did not differ with respect to the application of the most aggressive forms of respiratory support, frequency of transfer to ICU, and application of drugs. Detailed data are listed in Table 6.

### 3.3. Outcomes Depending on eGFR

There were statistically significant differences between groups A and B for all analyzed outcomes except for the number of days of hospitalization (Table 7). The highest difference in the percentage of deaths was recorded during hospitalization (10.36% in group A; 29.38% in group B). Out of all patients, 65.13% in group A and 38.15% in group B were discharged home. Patients with lower eGFR required emergency transfer to other specialist wards due to health deterioration or new health issues twice as frequently as patients with higher eGFR (20.29% vs. 11.18%).

Subgroups of patients classified by the level of eGFR below 60 mL/min/1.73 m^2^ varied by mortality during hospitalization, from the start to the 90th and 180th day (Table 8).

**Figure 1:**
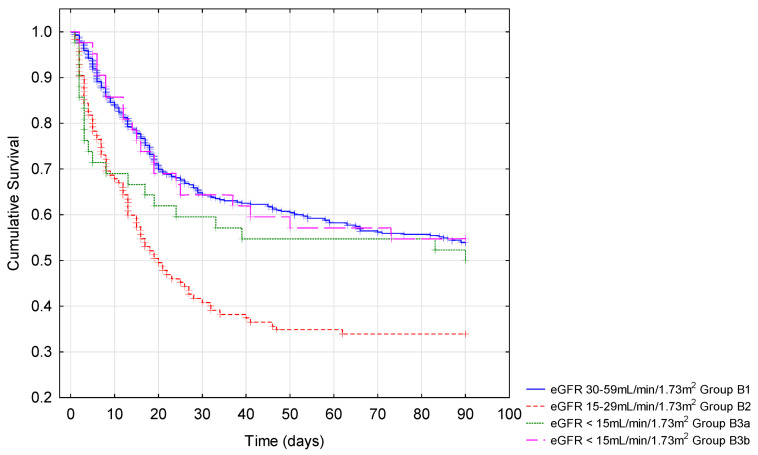
Kaplan–Meier cumulative survival analysis among patients with eGFR < 60 mL/min/1.73 m^2^ divided into four subgroups based on eGFR level at admission to hospital. Group B1: eGFR < 30–59 mL/min/1.73 m^2^; group B2: eGFR 15–29 mL/min/1.73 m^2^; group B3a: <15 mL/min/1.73 m^2^ without renal replacement therapy; group B3b < 15 mL/min/1.73 m^2^ on hemodialysis. Logarithmic rank analysis showed a statistical difference between the above mentioned four groups (*p* < 0.001). The lowest survival was found in B2 patients.

The highest percentage of deaths during hospitalization was recorded in subgroup B2 (44.44%) as compared to other subgroups (25.18, 32.56%, and 26.19% in subgroups B1, B3a, and B3b, respectively) (Figure 1).

Similarly, the highest mortality up to the 90th and 180th day concerned patients in the same subgroup. They were transferred to other specialist wards as an emergency more frequently than patients from other subgroups. No correlation was found between the level of eGFR and the number of days spent at the hospital.

### 3.4. Changes of Creatinine Concentration during Hospitalization

Creatinine concentration levels at admission and discharge were compared. A significant increase was defined as an increase in the creatinine concentration by 30% or more. In the entire studied group with COVID-19 (*n* = 1958), an increase in creatinine concentration was recorded in 145 patients (7.4%). In group A, creatinine concentration increased in 95 patients out of 1342 (7.08%), while in group B, it increased in 50 out of 616 (8.12%). The differences were not statistically significant (*p* = 0.415).

### 3.5. Occurrence of AKI during Hospitalization

In group A, AKI was observed in 118 patients (8.79%), while in group B, in 119 (19.32%). At admission, those patients in group A who would develop AKI during hospitalization differed significantly from non-AKI patients by gender (AKI in 10.7% of men and 6.65% of women), respiratory support (various forms of oxygen therapy in 73.73% of patients as compared to 42.44% in the non-AKI group), and comorbidities (hypertension in 67.8% and 39.71%, diabetes 32.2% and 17.09%, respectively). Detailed data are shown in Table 9.

Differences in lab parameters at admission between AKI and non-AKI patients concerned hemoglobin, lymphocytes, total protein, albumin (lower values in AKI group), as well as CRP, PCT, and IL-6 (higher concentrations in AKI group) (Table 10).

In the AKI group of patients, the COVID-19 course was more severe. Associated pneumonia was more frequent, and the patients were transferred to the ICU more frequently (48.31% vs. 5.8%, respectively). Further, a higher percentage of the patients required oxygen therapy, catecholamines, antibiotics, and intravenous administration of loop diuretics as compared to non-AKI patients. Especially, respiratory therapy was required for 50% of patients with AKI as compared to 5.47% in the non-AKI group (Table 9). During hospitalization, 62.71% of patients in the AKI group died, compared to 5.31% of patients without AKI (Table 11).

In group B, with lower eGFR, no difference in gender was found between patients with and without AKI; however, there were differences recorded at admission concerning the need for respiratory support and associated cardiovascular disorders. Other comorbidities, namely hypertension, diabetes, asthma, COPD, and liver diseases, were not observed significantly more frequently in any subgroup (Table 9). Patients with an impaired renal function who would develop AKI during hospitalization had lower concentrations of hemoglobin, total protein, slightly higher concentrations of creatinine, and significantly higher concentrations of PCT in admission lab tests than patients who would not develop AKI. There were no significant differences in IL-6 and CRP concentrations (Table 12).

In this population of COVID-19 patients, pneumonia developed in 71.43% of AKI patients and 54.73% of non-AKI patients. The former group was significantly more frequently on antibiotics, catecholamines, and intravenous loop diuretics. The percentage of patients transferred to the ICU was higher for the AKI group than for the non-AKI group: 36.97% and 7.85%, respectively (Table 9). Hospitalization ended with death in 60.5% patients with AKI and 21.93% of patients without AKI (Table 11).

Development of AKI was poorly associated with the studied predictors (clinical and laboratory variables), both in the group of patients with eGFR < 60 mL/min/1.73 m^2^ and the group of patients with eGFR ≥ 60 mL/min/1.73 m^2^. The adjusted R^2^ of the best logistic regression models was 0.09–0.10 and 0.13–0.16, respectively. Moreover, the *p*-values were larger than 0.05 for all the predictors.

**Figure 2:**
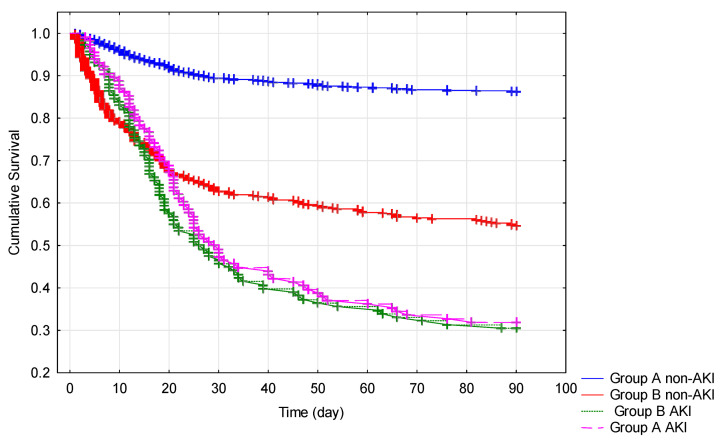
Kaplan–Meier cumulative survival analysis of COVID-19 patients until the 90th day from admission to hospital. All patients were divided into the four groups based on eGFR at admission and the AKI development during hospitalization. eGFR: Glomerular filtration rate. AKI: Acute kidney injury. Group A: eGFR ≥ 60 mL/min/1.73 m^2^; group B: eGFR < 60 mL/min/1.73 m^2^. Logarithmic rank analysis showed a statistical difference between the abovementioned four groups (*p* < 0.0000). AKI onset during hospitalization was associated with significantly higher mortality in COVID-19 patients independently from baseline eGFR.

Kaplan–Meier analysis (log-rank test) was used to compare survival from admission to hospital to the 90th day, with the patients divided into four groups based on whether patients had higher vs. lower eGFR at admission to hospital and whether they developed AKI or not during hospitalization. Logarithmic rank analysis showed a statistical difference in terms of survival between the four groups (*p* < 0.0001) (Figure 2). AKI onset during hospitalization was associated with significantly higher mortality in COVID-19 patients independently from baseline eGFR.

## 4. Discussion

Authors of numerous studies suggest that renal involvement in COVID-19 is common. Hallmarks of kidney injury such as elevated serum creatinine, urea and uric acid, hematuria, and proteinuria have been observed in up to 60% of affected patients [30,31,32]. Our study was an attempt at a comprehensive approach to renal function impairment in COVID-19 patients, considering bilateral associations in patients with significantly impaired renal function or not at admission.

In our study, 31.46% of patients with COVID-19 had eGFR < 60 mL/min/1.73 m^2^ at admission. Among them, 19.32% developed AKI during hospitalization. Further, AKI was diagnosed in 8.79% of COVID-19 patients with eGFR ≥ 60 mL/min/1.73 m^2^ at admission. Our study has also shown that COVID-19 is associated with the deterioration of kidney function. A comparison of the serum creatinine concentration at admission and discharge showed an increase in the creatinine concentration by 30% or more in 7.4% patients. These results not only confirm that kidney complications in COVID-19 are frequent regardless of the initial renal function, but they also highlight the necessity to monitor renal parameters in all COVID-19 patients and to ensure nephrological follow-up for some of them.

It is believed that there is a correlation between renal function impairment and increased risk of death in patients with COVID-19 [20,33,34,35]. In this study of nearly 2000 adults with COVID-19, we found that the mortality rate during hospitalization was much higher in patients with eGFR < 60 mL/min/1.73 m^2^ than in patients with eGFR ≥ 60 mL/min/1.73 m^2^ at admission (29.38% vs. 10.36%, respectively; *p* < 0.001). Analogically, the cumulative death rate up to the 90th and 180th days after admission was higher for patients with lower eGFR. It should be stressed that both groups of patients classified by the level of eGFR differed in all analyzed outcomes except for the number of days of hospitalization. Other than is mentioned above, among patients with eGFR < 60 mL/min/1.73 m^2^, health deterioration was observed in 39.12% of cases, and 13.47% of them had to be transferred to the ICU. In the group of patients with eGFR ≥ 60 mL/min/1.73 m^2^, the rates were 23.25% and 9.64% (*p* < 0.001), respectively. Our results are consistent with observations made by other authors [18,34,35,36]. In a UK population-based cohort study, increased risk of death was evident from eGFR 45–59 mL/min/1.73 m^2^ in people with type 1 and type 2 diabetes [37]. Gibertoni et al. revealed that non-dialyzed CKD patients had a 43.8% higher risk of dying than other hospitalized individuals with COVID-19 [36]. Meanwhile, Flythe et al. found that among COVID-19 patients with CKD treated at ICU, 50% of dialysis and non-dialysis-dependent patients died within 28 days after admission vs. 35% of patients without pre-existing CKD [31].

Researchers have pointed out the association between the specified level of kidney dysfunction among people with eGFR < 60 mL/min/1.73 m^2^ and mortality rate, but the respective data are not consistent [19,38]. Among patients with underlying kidney disease, those on maintenance dialysis had the highest risk of in-hospital mortality [17,31]. Results of the OpenSAFELY project documented that dialysis (adjusted hazard ratio (aHR) = 3.69) and the stage of CKD (aHR = 2.52 for patients with eGFR < 30 mL/min/1.73 m^2^) were two of the four comorbidity-related factors associated with the highest mortality risk in COVID-19 [39]. On the other hand, out of the 397 individuals with pre-existing non-dialysis-dependent CKD and known baseline serum creatinine levels, there was a nominally higher risk of in-hospital mortality with higher baseline serum creatinine concentrations, but the results did not reach statistical significance [31]. Further, in a study including 193 non-dialyzed CKD patients, the mortality rate increased gradually according to CKD stages (1–2, 3a,3b, 4): 25%, 28%, 39.73%, and 54.76%, respectively, decreasing in the fifth stage of CKD to 42.86% [36]. In our group of patients with eGFR < 60 mL/min/1.73 m^2^ at admission, the highest mortality was observed in the case of patients from the B2 subgroup with eGFR 15–29 mL/min/1.73 m^2^ (44.44%), which corresponds to CKD stage 4. This observation requires further analysis. The patients were close to “renal failure”, judging by the level of eGFR. In our study, patients from the B2 group were older than those from group B3. In addition, they had higher CRP, leukocytosis (only when compared to group B3b, not B3a), and lower serum albumin. The percentage of patients who developed pneumonia during hospitalization, needed respiratory therapy, and were transferred to ICU was higher in group B2 compared to group B3. The above observations may suggest the relation of survival with pneumonia, respiratory system sufficiency, and non-modifiable factors such as age.

In our study, the percentage of patients with cardiovascular disorders, diabetes mellitus, and COPD was higher in the group with lower eGFR and a higher mortality rate. Analogical findings were reported by other authors [33]. Ozturk et al. observed that CKD patients (without RRT) had higher mortality than HD patients and the highest mortality of CKD patients was attributed to the high burden of cardiovascular comorbidities [19]. Among patients who required ICU treatment, associated conditions including diabetes and cardiovascular disorders were more common in patients with pre-existing CKD compared to those without CKD, too [31]. In a study concerning patients with COPD and COVID-19, Bonato et al. showed worse outcomes in patients with the presence of emphysema and low DLCO (diffusing capacity of the lung for carbon monoxide) [40]. There are also interesting data concerning hypertension. In our study, patients from groups A and B differed in the incidence of hypertension. The percentage of patients with hypertension was higher in the group with eGFR < 60 mL/min/1.73 m^2^ (group B) than among patients with higher eGFR (68.83% vs. 42.18%, respectively; *p* < 0.001); however, no differences between the B1-B3 subgroups were observed (*p* = 0.08). Findings by other authors are varied [18,32]. Some argue that hypertension may rather form a background for other clinical conditions that increase the risk of severe COVID-19 in patients with kidney dysfunction [24]. On the other hand, a comparative analysis of a large cohort of patients with a history of arterial hypertension (*n* = 2850) and without (*n* = 2960) [HOPE Registry (Health Outcome Predictive Evaluation for COVID-19)] indicated that the mortality rate and in-hospital complications might be increased in COVID-19 patients with a concomitant history of arterial hypertension [41]. The reported mortality rate was 29.6% in patients with associated arterial hypertension and 11.3% in those without arterial hypertension [41]. However, the group of patients with hypertension also had a higher rate of comorbidities, among others heart disease, diabetes mellitus, lung disease, and kidney disease with creatinine clearance < 30 mL/min compared to the group of patients without hypertension (36.5%, 29.8%, 24%, and 11.5% vs. 9.8%, 7.8%, 13.6%m and 1.7%) [41]. In general, the presence of comorbidities has been described as a factor affecting COVID-19 outcomes [32,40,42,43]. The literature shows that comorbidities are not only among factors associated with the risk of death, but that they are also a risk factor for development of COVID-19 [36,39]. Many studies have shown that patients who developed COVID-19 had a higher prevalence of hypertension, type-2 diabetes, obesity, and hyperlipidemia than those who did not [33,40]. In a meta-analysis by Peiris et al., the four most frequently reported comorbidities were arterial hypertension (40.8%), diabetes mellitus (22%), cardiovascular disease (17.2%), and obesity (11.5%) [6].

A search for reasons of the increased mortality of patients with impaired renal function must not omit immunologic disorders. The deterioration of kidney function per se is associated with a proinflammatory state [44,45]. In our present study, in the group of patients with eGFR < 60 mL/min/1.73 m^2^ CRP, PCT, and IL-6 concentrations were higher than in the group with better kidney function. IL-6 is a multifunctional cytokine that transmits cell signaling and regulates immune cells. IL-6, which is promptly and transiently produced in response to infections and tissue injuries, contributes to host defense by stimulating acute phase responses, hematopoiesis, and immune reactions [46]. CRP is an acute-phase proinflammatory cytokine and a sensitive biomarker of infection and tissue damage [47]. Numerous studies have shown that patients with poorer kidney function (eGFR < 60 mL/min/1.73 m^2^) at admission presented increased inflammatory biomarker values (such as CRP, PCT, and ferritin) compared to individuals without significant kidney deterioration (eGFR > 60 mL/min/1.73) [48]. Secreting CRP and/or IL-6 always induces a cytokine storm and impairs the functioning of multiple organs. Moreover, patients with renal dysfunction display an increased risk of infections compared to people with better kidney function [45]. In our present study, patients from group B suffered from pneumonia more frequently than those in group A (57.95% vs. 51.04%; *p* = 0.004). In more patients in this group antibiotic therapy was needed and more intensive forms of ventilation support were required. A higher frequency of pneumonia may be associated with a dysregulated immune response in patients with kidney deterioration. An analysis of the international Health Outcome Predictive Evaluation for COVID-19 registry (HOPE COVID-19) evaluating the impact of renal function at admission (eGFR > 60 mL/min/1.73 m^2^, eGFR 30–60 mL/min/1.73 m^2^, and eGFR < 30 mL/min/1.73 m^2^) on outcomes and mortality in 758 patients with SARS-CoV-2 infection revealed that patients with kidney dysfunction at hospital admission presented a higher incidence of complications such as sepsis (11.9% vs. 26.4% vs. 40.8%, respectively; *p* < 0.001) and respiratory failure (35.4% vs. 72.2% vs. 62.0%, respectively; *p* < 0.001) [49]. However, the occurrence of pneumonia seems to be multifactorial. In our study, the analysis of incidence of pneumonia in the group of patients with impaired renal function classified by the level of eGFR showed the highest percentage of pneumonia in patients with eGFR 30–59 mL/min/1.73 m^2^ as compared to those with even lower levels of eGFR < 30 mL/min/1.73 m^2^. This result was probably affected by additional factors. Immune dysfunction has been described in the population with kidney impairment, but it may be manifested either by immunosuppression or overactivation [44]. It cannot be ruled out that patients with various levels of kidney function deterioration do not display the same cytokine storm or inflammatory response implicated in diverse clinical manifestations [1]. Liu et al. performed a systematic review and meta-analysis to verify if pre-existing CKD was a risk factor for severe COVID-19 [34]. According to their findings, there was no significant difference with respect to CKD between the group with critical and severe courses of COVID-19. The authors speculated that the cytokine storm might be weakened by CKD itself and that these patients were not vulnerable to critical COVID-19 because of immunodepression due to CKD. Unfortunately, the analyzed studies did not refer to the stage of CKD, which would add important information.

Literature data indicate respiratory failure, septic shock, and kidney failure as the main causes of death in COVID patients [50]. In this context, AKI may play an important role for the outcomes of patients with COVID [9,51]. It is regarded as a common complication of COVID-19 and it can develop independently of baseline kidney function [7,9,35]. However, kidney impairment has been recognized as a risk factor for AKI [9,16]. Other risk factors for AKI in COVID-19 patients include diabetes mellitus, hypertension, male gender, the need for ventilation support, and high interleukin-6 levels [10,50]. Our observations were partially consistent with the described findings. The outcomes correlated with eGFR at admission to hospital. In our present study, AKI prevalence was higher in the group of patients with lower eGFR as compared to the group with higher eGFR (19.32% vs. 8.79%, respectively; *p* < 0.001), which might have contributed to excess mortality in patients with baseline kidney impairment. In the present study, those patients who did not have serious renal impairment but who developed AKI (8.79% in group A) differed significantly from patients without AKI by gender (more frequently male patients), comorbidities (more frequently diabetes mellitus and hypertension in medical history), and the need for respiratory support at admission (various forms of oxygen therapy in 73.73% vs. 42.44% of the patients). Moreover, they had higher CRP, PCT, and IL-6 levels. Meanwhile, patients with impaired renal function at admission who developed AKI (19.32% in group B) also needed ventilation support at admission more frequently (56.3% vs. 52.42%), but gender, the presence of hypertension and diabetes, CRP, and IL-6 levels were not different compared to patients from group B without AKI. However, they displayed a higher rate of cardiac diseases. In our study, none of the analyzed demographic, clinical, or laboratory variables observed at admission were proven to be significant predictors of AKI during hospitalization. On the other hand, our analysis showed that AKI was a significant determining factor of mortality in COVID-19, regardless of the initial renal function, as shown by Kaplan–Meier survival curves. AKI was associated with death within 90 days of follow-up: 68.10% patients with eGFR ≥ 60 mL/min/1.73 m^2^ and 69.10% patients with eGFR < 60 mL/min/1.73 m^2^ at admission. Similar findings were reported by Kang et al. [33]. Their study of 7341 COVID-19 patients, but with a lower number of CKD individuals (*n* = 253) than in our cohort, showed that AKI was more closely associated with adverse outcomes than CKD. It can be suggested that AKI in the course of COVID-19 (COV-AKI) is connected to general conditions in which kidneys are involved [6]. COV-AKI may be a result of indirect hemodynamic and immunologic effects rather than direct kidney injury [5].

Our data suggest that COVID-19 may have induced the onset or progression of renal dysfunction. An increase in the creatinine concentration at discharge by 30% or more as compared to the concentration at admission was observed in 7.4% of patients. The percentage was similar in both groups of patients classified by eGFR at admission (<60 mL/min/1.73 m^2^ vs. ≥60 mL/min/1.73 m^2^), reaching 7.08% and 8.12%, respectively. These results are consistent with findings by Kellum et al. [7], as well as Xiang [32]. The negative impact of COVID-19 on renal function was also identified in studies concerning patients who developed AKI during hospitalization [8,9]. It was found that among patients with AKI who were discharged from the hospital, 35% had not recovered to baseline kidney function by the time of discharge [9]. Similar findings were reported by Pei et al. [52]. In their study of a Chinese cohort, in fewer than half of patients, a full recovery of kidney function was observed. These results may suggest negative consequences of COV-AKI for affected people, such as post COVID-19 acute kidney disease, CKD, or progression of prior CKD. The percentage of our patients who had been vaccinated against SARS-CoV-2 was small. Given the high possibility of kidney dysfunction in COVID-19, in order to reduce the incidents and severity of this disease, the vaccination should be implemented as soon as possible, especially in patients with impaired renal function.

Our study had certain limitations. This was a retrospective observational study, and the groups were not randomized. Some clinical and laboratory variables were missing for many patients. Several data were not included in the analysis. Next, we categorized patients into two main groups based on eGFR at admission: With eGFR ≥ 60 mL/min/1.73 m^2^ and eGFR < 60 mL/min/1.73 m^2^, because a decrease in eGFR is a good marker of deterioration of kidney function. It cannot be excluded that the coincidence of true kidney dysfunction and lower eGFR may be temporary. However, the high percentage of patients with diabetes and hypertension makes chronic renal impairment with eGFR < 60 mL/min/1.73 m^2^ highly probable in many patients. We considered history data concerning prior CKD; however, it is well known CKD is underestimated, and a lack of diagnosis of chronic kidney dysfunction at admission to hospital appears to be an unsuitable criterion for its exclusion. Moreover, we did not have pre-hospital serum creatinine data, which are infrequently routinely available. The definition of AKI during hospitalization was based only on one condition, i.e., the increase in serum creatinine. Zhou et al. found that severe COV-AKI seemed to develop at a median of 15 days (IQ 13–19.5 days) [53], so it may be speculated that the lower eGFR at admission was a result of chronic kidney dysfunction rather than AKI. The period of the patients’ inclusion in the study was long, covering both the first and second wave of the pandemic disease. This might have affected the demographic and clinical characteristics of patients admitted to the hospital, and the choice of treatment, adequately to the changing knowledge about the specific course of COVID-19, and finally the mortality rate [54]. On the other hand, the study covered a large group of patients, analyzing not only in-hospital mortality, but also deaths within 90 and 180 days from admission to hospital, which may increase the strength of the obtained results.

Our study included a population from one country. Thus, different phenotypes and genotypes that may influence the clinical manifestation and laboratory findings and evolution have been excluded.

Concluding, in this single-center study of almost 2000 adult patients with COVID-19 confirmed with a PCR test, we have found that the course of COVID-19 in the group of patients with impaired renal function at admission evidenced by the level of eGFR < 60 mL/min/1.73 m^2^ is worse than in patients with initial eGFR > 60 mL/min/1.73 m^2^. The higher mortality of patients with lower eGFR seems associated with comorbidities, respiratory dysfunctions, immunological impairment, and more frequent development of AKI during hospitalization. The occurrence of AKI is a factor of significant negative impact on patients’ survival, regardless of the initial renal function. In 7–8% of COVID-19 patients, a significant deterioration of renal function was observed in the follow-up period to the end of hospitalization. Our results highlight the need for monitoring kidney parameters in all COVID-19 patients for eliminating and treating conditions stimulating the development of AKI and ensuring further nephrology care for some patients after COVID-19. In future research on the association between renal function and COVID-19, it seems important to determine predictors of AKI development and deterioration of renal function as observed at the onset of the disease and/or at hospital admission. Further studies should also focus on defining a “phenotype” of unfavorable prognosis in patients with COVID-19 and reduced eGFR, depending on its value.

## Figures and Tables

**Table 1:** Baseline characteristics of COVID-19 patients at admission to hospital.

Variables	Group A (*n* = 1342) eGFR ≥ 60 mL/min/1.73		Group B (*n* = 616) eGFR < 60 mL/min/1.73		
	*n*	*%*	*n*	*%*	*p*-Value
Gender					0.319
women	632	47.09	305	49.51	
men	710	52.91	311	50.49	
Age (years) *	58 ± 17.49		71.79 ± 13.56		0.01
Vaccination ^1^					0.869
no	917	95.62	411	95.80	
one dose	35	3.65	14	3.26	
two	7	0.73	4	0.93	
Respiratory support					0.001
no	735	54.81	327	53.17	
oxygen mustache cannula	313	23.34	106	17.24	
face mask	89	6.64	54	8.78	
Venturi mask	9	0.67	8	1.30	
passive oxygen therapy	134	9.99	81	13.17	
HFNC	3	0.22	7	1.14	
BiPAP/CPAP	5	0.37	4	0.65	
respiratory therapy	53	3.95	28	4.55	
Hypertension					<0.001
no	776	57.82	192	31.17	
yes	566	42.18	424	68.83	
Diabetes mellitus					<0.001
no	1094	81.58	364	59.19	
type 1	13	0.97	18	2.93	
type 2 (oral therapy)	158	11.78	148	24.07	
type 2 (insulin therapy)	50	3.73	73	11.87	
prediabetes	20	1.49	12	1.95	
Cardiovascular disease					<0.001
no	1245	92.77	462	75.00	
yes	97	7.23	154	25.00	
Asthma					0.356
no	1282	95.53	594	96.43	
yes	60	4.47	22	3.57	
COPD					<0.001
no	1306	97.32	578	93.83	
yes	36	2.68	38	6.17	
Liver diseases					0.944
no	1294	96.50	594	96.43	
chronic hepatitis ^2^	17	1.27	8	1.30	
cirrhosis with portal hypertension	8	0.60	5	0.81	
steatosis (NASH/NAFLD)	22	1.64	9	1.46	
Smoking					0.082
no	1223	91.27	541	88.11	
in the past	70	5.22	46	7.49	
now	47	3.51	27	4.40	
Home medication					
ACEI					<0.001
no	1147	85.47	461	74.84	
yes	195	14.53	155	25.16	
ARB					0.798
no	1244	92.70	573	93.02	
yes	98	7.30	43	6.98	
Beta-blocker					<0.001
no	1070	79.73	366	59.42	
yes	272	20.27	250	40.58	
CCB					<0.001
no	1221	90.98	480	77.92	
yes	121	9.02	136	22.08	
Alfa-blocker					<0.001
no	1288	95.98	553	89.77	
yes	54	4.02	63	10.23	
Statin					<0.001
no	1163	86.66	446	72.40	
yes	179	13.34	170	27.60	
Loop diuretic					<0.001
no	1277	95.16	497	80.68	
yes	65	4.84	119	19.32	
Thiazides or thiazide-like diuretics					0.019
no	1254	93.44	557	90.42	
yes	88	6.56	59	9.58	

eGFR—estimated glomerular filtration rate; HFNC—High Flow Nasal Cannula; BiPAP/CPAP—Bilevel Positive Airway Pressure/Continuous Positive Airway Pressure; COPD—chronic obstructive pulmonary disease; NASH—non-alcoholic steatohepatitis; NAFLD—non-alcoholic fatty liver disease; ACEI—angiotensin-converting-enzyme inhibitor; ARB—angiotensin II receptor blocker; CCB—calcium channel blocker. ^1^ vaccination against SARS-CoV-2; ^2^ or cirrhosis without portal hypertension. Values were presented as number of observations (percent) and * mean ± SD.

**Table 2:** Laboratory values of COVID-19 patients at admission to hospital.

	A Group (*n* = 1342) eGFR ≥ 60 mL/min/1.73 m^2^	B Group (*n* = 616) eGFR < 60 mL/min/1.73 m^2^	*p*-Value
HGB, g/dL	13.5 (2.5)	12.50(3.45)	≤0.0001
WBC, 10^3^/µL	7.09 (4.56)	8.06 (6.03)	≤0.0001
Lymphocytes, 10^3^/µL	1.01 (0.78)	0.88 (0.78)	≤0.0001
PLT, 10^3^/µL	215.00 (120.00)	200.50 (119.00)	0.0004
Albumin, g/dL	3.20 (0.80)	3.00 (0.80)	≤0.0001
Total protein, g/dL	6.10 (1.10)	5.80 (1.10)	0.0001
CRP, mg/dL	44.23 (94.98)	67.89 (120.94)	≤0.0001
D-dimer, µg/mL	1.03 (1.99)	1.79 (4.36)	≤0.0001
Fibrinogen, g/L	4.76 (2.30)	4.60 (2.64)	0.492
IL-6, pg/mL	15.30 (36.52)	24.70 (50.26)	0.0002
Uric acid, mg/dL	4.80 (2.10)	7.05 (3.35)	≤0.0001
Creatinine, mg/dL	0.82 (0.24)	1.70 (1.16)	≤0.0001
Urea, mg/dL	31.00 (20.00)	76.00 (57.00)	≤0.0001
Potassium, mmol/L	4.00 (0.67)	4.30 (0.93)	≤0.0001
PCT, ng/mL	0.06 (0.13)	0.27 (0.84)	≤0.0001
Sodium, mmol/L	138.00 (5.00)	138.00 (6.00)	0.361
INR	1.10 (0.19)	1.17 (0.27)	≤0.0001
APTT	32.00 (8.20)	33.10 (9.90)	0.0003

eGFR: Estimated glomerular filtration rate; HGB: Hemoglobin; WBC: White blood cells; PLT: Platelets; CRP: C reactive protein; IL-6: interleukin-6; PCT: Procalcitonin; INR: International normalized ratio; APTT: Activated partial thromboplastin time. Data are given as median and interquartile range.

**Table 3:** Characteristics of COVID-19 patients with eGFR < 60 mL/min/1.73 m^2^ (group B) divided into subgroups according to eGFR value at admission.

Variables	Group B1 eGFR 59-30 (*n* = 409)		Group B2 eGFR 29-15 (*n* = 122)		^a^ Group B3a eGFR < 15 m^2^ (*n* = 43)		^b^ Group B3b eGFR < 15 (*n* = 42)		*p*-Value
	*n*	*%*	*n*	*%*	*n*	*%*	*n*	%	
Gender									
women	199	48.66	67	54.92	24	55.81	15	35.71	0.143
men	210	51.34	55	45.08	19	44.19	27	64.29	
Age (years) *	72.48 ± 13.07		73.38 ± 12.65		66.81 ± 17.49		65.55 ± 14.00		0.01
Vaccination ^1^									0.732
no	270	95.41	83	95.40	30	100.00	28	96.55	
one dose	9	3.18	4	4.60	0	0.00	1	3.45	
two	4	1.41	0	0.00	0	0.00	0	0.00	
Respiratory support									0.227
no	208	50.98	62	50.82	31	72.09	26	61.90	
oxygen mustache cannula	78	19.12	17	13.93	2	4.65	9	21.43	
face mask	35	8.58	14	11.48	2	4.65	3	7.14	
Venturi mask	4	0.98	3	2.46	0	0.00	1	2.38	
passive oxygen therapy	51	12.50	20	16.39	7	16.28	3	7.14	
HFNC	6	1.47	0	0.00	1	2.33	0	0.00	
BiPAP/CPAP	3	0.74	1	0.82	0	0.00	0	0.00	
respiratory therapy	23	5.64	5	4.10	0	0.00	0	0.00	
Hypertension									0.082
no	133	32.52	37	30.33	16	37.21	6	14.29	
yes	276	67.48	85	69.67	27	62.79	36	85.71	
Diabetes mellitus									0.405
no	250	61.27	66	54.10	25	58.14	23	54.76	
type 1	7	1.72	6	4.92	3	6.98	2	4.76	
type 2 (oral therapy)	98	24.02	34	27.87	9	20.93	7	16.67	
type 2 (insulin therapy)	43	10.54	15	12.30	6	13.95	9	21.43	
prediabetes	10	2.45	1	0.82	0	0.00	1	2.38	
Cardiovascular disease									0.007
no	319	78.00	80	65.57	36	83.72	27	64.29	
yes	90	22.00	42	34.43	7	16.28	15	36.71	
Asthma:									0.750
no	392	95.84	119	97.54	42	97.67	41	97.62	
yes	17	4.16	3	2.46	1	2.33	1	2.38	
COPD									0.358
no	383	93.64	113	92.62	43	100.00	39	92.86	
yes	26	6.36	9	7.38	0	0.00	3	7.14	
Liver diseases									0.630
no	394	96.33	118	96.72	42	97.67	40	95.24	
chronic hepatitis ^2^	4	0.98	2	1.64	0	0.00	2	4.76	
cirrhosis with portal hypertension	4	0.98	1	0.82	0	0.00	0	0.00	
steatosis (NASH/NAFLD)	7	1.71	1	0.82	1	2.33	0	0.00	
Smoking									0.876
no	358	87.96	106	86.89	40	93.02	37	88.10	
in the past	32	7.86	10	8.20	2	4.65	2	4.76	
now	17	4.18	6	4.92	1	2.33	3	7.14	
Home medication									
ACEI									0.022
no	291	71.15	103	84.43	34	79.07	33	78.57	
yes	118	28.85	19	15.57	9	20.93	9	21.43	
ARB									0.01
no	371	90.71	117	95.90	43	100.00	42	100.00	
yes	38	9.29	5	4.10	0	0.00	0	0.00	
Beta-blocker									<0.001
no	251	61.37	73	59.84	30	69.77	12	28.57	
yes	158	38.63	49	40.16	13	30.23	30	71.43	
CCB									<0.001
no	323	78.97	103	84.43	34	79.07	20	47.62	
yes	86	21.03	19	15.57	9	20.93	22	52.38	
Alfa-blocker									<0.001
no	383	93.64	109	89.34	39	90.70	22	52.38	
yes	26	6.36	13	10.66	4	9.30	20	47.62	
Statin									0.094
no	292	71.39	88	72.13	38	88.37	28	66.67	
yes	117	28.61	34	27.87	5	11.63	14	33.33	
Loop diuretic									<0.001
no	342	83.62	95	77.87	38	88.37	22	52.38	
yes	67	16.38	27	22.13	5	11.63	20	47.62	
Thiazides or thiazide-like diuretics									0.036
no	361	88.26	113	92.62	41	95.35	42	100.00	
yes	48	11.74	9	7.38	2	4.65	0	0.00	

eGFR—estimated glomerular filtration rate (mL/min/1.73 m^2^); HFNC—High Flow Nasal Cannula; BiPAP/CPAP—Bilevel Positive Airway Pressure/Continuous Positive Airway Pressure; COPD—chronic obstructive pulmonary disease; NASH—non-alcoholic steatohepatitis; NAFLD—non-alcoholic fatty liver disease; ACEI—angiotensin-converting-enzyme inhibitor; ARB—angiotensin II receptor blocker; CCB—calcium channel blocker. ^1^ vaccination against SARS-CoV-2; ^2^ or cirrhosis without portal hypertension; ^a^ patients without RRT (renal replacement therapy); ^b^ patients on hemodialysis. Values were presented as number of observations (percent) and * mean ± SD.

**Table 4:** Laboratory values of patients with eGFR < 60 mL/min/1.73 m^2^ grouped according to eGFR value at admission.

	Group B1 eGFR 59-30 (*n* = 409)	Group B2 eGFR 29-15 (*n* = 122)	^a^ Group B3a eGFR < 15 (*n* = 43)	^b^ Group B3b eGFR < 15 (*n* = 42)	*p*-Value
HGB, g/dL	12.80 (3.10)	12.10 (3.50)	10.20 (3.70)	10.00 (2.70)	≤0.0001
WBC, 10^3^/µL	7.95 (5.18)	10.07 (7.68)	10.93 (8.20)	5.14 (3.47)	≤0.0001
Lymphocytes, 10^3^/µL	0.90 (0.77)	0.90 (0.78)	0.83 (0.98)	0.70 (0.52)	0.118
PLT, 10^3^/µL	208.00 (118.00)	199.00 (130.00)	224.00 (179.00)	163.00 (67.00)	0.0032
Albumin, g/dL	3.00 (0.70)	2.80 (0.70)	3.00 (0.70)	3.15 (0.65)	0.0424
Total protein, g/dL	5.95 (1.20)	5.70 (1.10)	5.80 (1.15)	5.95 (1.35)	0.481
CRP, mg/dL	66.52 (116.13)	90.16 (142.53)	87.30 (117.91)	37.90 (79.45)	0.0011
D-dimer, µg/mL	1.77 (4.33)	1.40 (5.57)	0.94 (1.83)	2.68 (11.24)	0.494
Fibrinogen, g/L	4.66 (2.43)	4.83 (3.04)	3.44 (1.01)	5.27 (2.85)	0.425
IL-6, pg/mL	22.05 (46.50)	28.35 (51.90)	66.25 (499.90)	39.80 (42.15)	0.069
Uric acid, mg/dL	7.00 (3.10)	7.00 (3.60)	10.10 (4.70)	5.80 (2.10)	0.0004
Creatinine, mg/dL	1.40 (0.50)	2.52 (0.94)	5.16 (2.19)	5.72 (2.31)	≤0.0001
Urea, mg/dL	64.00 (38.00)	118.00 (60.00)	180.00 (91.00)	109.00 (80.00)	≤0.0001
Potassium, mmol/L	4.20 (0.80)	4.40 (1.00)	4.62 (1.10)	5.09 (1.10)	≤0.0001
PCT, ng/mL	0.17 (0.51)	0.53 (2.32)	0.68 (1.03)	0.56 (1.42)	≤0.0001
Sodium, mmol/L	138.00 (6.00)	138.00 (7.00)	138.50 (9.00)	136.50 (4.00)	0.092
INR	1.15 (0.24)	1.21 (0.40)	1.24 (0.33)	1.13 (0.21)	0.0010
APTT	32.90 (10.10)	32.90 (8.90)	35.50 (8.20)	33.40 (12.00)	0.56

eGFR—estimated glomerular filtration rate (mL/min/1.73 m^2^); ^a^ patients without RRT (renal replacement therapy); ^b^ patients on hemodialysis; HGB: hemoglobin; WBC: white blood cells; PLT: platelets; CRP: C reactive protein; IL-6: interleukin-6; PCT: procalcitonin; INR: international normalized ratio; APTT: activated partial thromboplastin time. Data are given as median and interquartile range.

**Table 5:** Comparison of clinical parameters during hospitalization between two groups of COVID-19 patients.

Variables	Group A (*n* = 1342) eGFR ≥ 60 mL/min/1.73		Group B (*n* = 616) eGFR < 60 mL/min/1.73		*p*-Value
	*n*	*%*	*n*	*%*	
Pneumonia					0.004
no	657	48.96	259	42.05	
yes	685	51.04	357	57.95	
Deterioration of patient’s condition					<0.001
no	1030	76.75	375	60.88	
yes	312	23.25	241	39.12	
The most aggressive respiratory support					0.003
without oxygen therapy	585	43.59	242	39.48	
passive low-flow oxygen therapy	523	38.97	223	36.38	
passive high-flow oxygen therapy	87	6.48	44	7.18	
non-invasive ventilation (BiPAP/CPAP)	21	1.56	21	3.43	
respiratory therapy	126	9.39	83	13.54	
Transfer to ICU					0.009
no	1214	90.46	533	86.53	
yes	128	9.54	83	13.47	
Antibiotics					<0.001
no	577	43.00	188	30.52	
yes	765	57.00	428	69.48	
Loop diuretics i.v.					<0.001
no	1171	87.26	455	73.86	
yes	171	12.74	161	26.14	
Catecholamines					<0.001
no	1225	96.61	517	90.86	
yes	43	3.39	52	9.14	

eGFR—estimated glomerular filtration rate; BiPAP/CPAP—Bilevel Positive Airway Pressure/Continuous Positive Airway Pressure; ICU—Intensive Care Unit. Values were presented as number of observations (percent).

**Table 6:** Comparison of clinical parameters during hospitalization between subgroups of group B depending on eGFR value.

Variables	Group B1 eGFR 59-30 (*n* = 409)		Group B2 eGFR 29-15 (*n* = 122)		^a^ Group B3a eGFR < 15 (*n* = 43)		^b^ Group B3b eGFR < 15 (*n* = 42)		*p*-Value
	*n*	*%*	*n*	*%*	*n*	*%*	*n*	%	
Pneumonia									0.04
no	159	38.88	55	45.08	26	60.47	19	45.24	
yes	250	61.12	67	54.92	17	39.53	23	54.76	
Deterioration of patient’s condition									0.058
no	259	63.33	63	51.64	30	69.77	23	54.76	
yes	150	36.67	59	48.36	13	30.23	19	45.24	
The most aggressive respiratory support									0.256
without oxygen therapy	158	38.82	46	37.70	23	53.49	15	36.59	
passive low-flow oxygen therapy	148	36.36	40	32.79	14	32.56	21	51.22	
passive high-flow oxygen therapy	29	7.13	11	9.02	2	4.65	2	4.88	
non-invasive ventilation (BiPAP/CPAP)	12	2.95	7	5.74	2	4.65	0	0.00	
respiratory therapy	60	14.74	18	14.75	2	4.65	3	7.32	
Transfer to ICU									0.115
no	350	85.57	103	84.43	42	97.67	38	90.48	
yes	59	14.43	19	15.57	1	2.33	4	9.52	
Antibiotics									0.071
no	135	33.01	28	22.95	16	37.21	9	21.43	
yes	274	66.99	94	77.05	27	62.78	33	78.57	
Loop diuretics i.v.									0.277
no	309	75.55	83	68.03	34	79.07	29	69.05	
yes	100	24.45	39	31.97	9	20.93	13	30.95	
Catecholamines									0.286
no	344	84.11	97	79.51	39	90.70	37	88.10	
yes	65	15.89	25	20.49	4	9.30	5	11.90	

eGFR—estimated glomerular filtration rate(mL/min/1.73 m^2^); BiPAP/CPAP—Bilevel Positive Airway Pressure/Continuous Positive Airway Pressure; ICU—Intensive Care Unit. ^a^ patients without RRT (renal replacement therapy); ^b^ patients on hemodialysis. Values were presented as number of observations (percent).

**Table 7:** Comparison of outcomes between two tested groups of COVID patients.

Variables	Group A (*n* = 1342) eGFR ≥ 60 mL/min/1.73		Group B (*n* = 616) eGFR < 60 mL/min/1.73		*p*-Value
	*n*	*%*	*n*	*%*	
In-hospital mortality					<0.001
no	1203	89.64	435	70.62	
yes	139	10.36	181	29.38	
Death within 90 days of admission					<0.001
no	1037	81.40	296	49.92	
yes	237	18.60	297	50.08	
Death within 180 days of admission					<0.001
no	306	54.64	144	31.65	
yes	254	45.36	311	68.35	
End of hospitalization					<0.001
discharge home	874	65.13	235	38.15	
emergency transfer to other centers	150	11.18	125	20.29	
transfer to other centers for rehabilitation	179	13.34	75	12.18	
death	139	10.36	181	29.38	
Number of hospitalization days ^1^	10 (2–16)		10 (2–19)		0.36

eGFR—estimated glomerular filtration rate. Values were presented as number of observations (percent). ^1^ median and interquartile range.

**Table 8:** Comparison of outcomes between subgroups of group B (eGFR < 60 mL/min/1.73 m^2^).

Variables	Group B1 eGFR 59-30 (*n* = 409)		Group B2 eGFR 29-15 (*n* = 122)		^a^ Group B3a eGFR < 15 (*n* = 43)		^b^ Group B3b GFR < 15 (*n* = 42)		*p*-Value
	*n*	*%*	*n*	*%*	*n*	*%*	*n*	%	
In-hospital mortality									0.001
no	306	74.82	69	56.56	29	67.44	31	73.81	
yes	103	25.18	53	53.44	14	32.56	11	26.19	
Death within 90 days of admission									0.002
no	213	53.92	39	33.91	21	50.00	23	54.76	
yes	182	46.08	76	66.09	21	50.00	19	45.24	
Death within 180 days of admission									0.002
no	98	34.03	16	16.67	14	38.89	16	44.44	
yes	190	65.97	80	83.33	22	61.11	20	55.56	
End of hospitalization									0.007
discharge home	173	42.30	30	24.59	14	32.56	18	42.86	
emergency transfer to other centers	83	20.29	28	22.95	8	18.60	6	14.29	
transfer to other centers for rehabilitation	50	12.22	11	9.02	7	16.28	7	16.67	
death	103	25.18	53	43.44	14	32.56	11	26.19	
Number of hospitalization days ^1^	10 (2–19)		8 (2–18)		9 (2–14)		14.5 (6–20)		0.119

eGFR—estimated glomerular filtration rate (mL/min/1.73 m^2^). ^a^ patients without RRT (renal replacement therapy); ^b^ patients on hemodialysis. Values were presented as number of observations (percent). ^1^ median and interquartile range.

**Table 9:** AKI in COVID-19 patients: Comparison of two groups.

Variables	Group A (eGFR ≥ 60)					Group B (eGFR < 60)				
	Non-AKI (*n* = 1224)		AKI (*n* = 118)		*p*-Value	Non-AKI (*n* = 497)		AKI (*n* = 119)		*p*-Value
	*n*	*%*	*n*	*%*		*n*	*%*	*n*	%	
Baseline characteristics at admission										
Gender					0.009					0.315
women	590	48.20	42	35.59		251	50.50	54	45.38	
men	634	51.80	76	64.41		246	49.50	65	54.62	
Age (years) *	57.32 ± 17.65		65.13± 17.65		0.001	70.14 ± 13.28		70.32 ± 14.66		0.3
Vaccination ^1^					0.460					0.485
no	823	95.37	94	97.92		324	95.29	87	97.75	
one dose	33	3.82	2	2.08		12	3.53	2	2.25	
two	7	0.81	0	0		4	1.18	0	0.00	
Respiratory support					<0.001					<0.001
no	704	57.56	31	26.27		260	52.42	67	56.30	
oxygen mustache cannula	290	23.71	23	19.49		95	19.15	11	9.24	
face mask	76	6.21	13	11.02		45	9.07	9	7.56	
Venturi mask	8	0.65	1	0.85		8	1.61	0	0.00	
passive oxygen therapy	111	9.08	23	19.49		67	13.51	14	11.76	
HFNC	1	0.08	2	1.96		5	1.01	2	1.68	
BiPAP/CPAP	1	0.08	4	3.39		2	0.40	2	1.68	
respiratory therapy	32	2.62	21	17.80		14	2.82	14	11.76	
Hypertension					<0.001					0.367
no	738	60.29	38	32.20		159	31.99	33	27.73	
yes	486	39.71	80	67.80		338	68.01	86	72.27	
Diabetes mellitus					<0.001					0.741
no	1014	82.91	80	67.80		299	60.28	65	54.62	
type 1	13	1.06	0	0.00		13	2.62	5	4.20	
type 2 (oral therapy)	130	10.63	28	23.73		13	2.62	5	4.20	
type 2 (insulin therapy)	43	3.52	7	5.93		118	23.79	30	25.21	
prediabetes	17	1.39	3	2.54		57	11.49	16	13.45	
Cardiovascular disease					0.196					<0.001
no	1139	93.06	106	89.83		387	77.87	75	63.03	
yes	85	6.94	12	10.17		110	22.13	44	36.97	
Asthma					0.898					0.680
no	1169	95.51	113	95.76		478	96.18	116	97.48	
yes	55	4.49	5	4.24		19	3.82	3	2.52	
COPD					0.09					0.780
no	1194	97.55	112	94.92		467	93.96	111	93.28	
yes	30	2.45	6	5.08		30	6.04	8	6.72	
Liver diseases					0.146					0.114
no	1181	96.57	113	95.76		480	96.58	114	95.80	
chronic hepatitis ^2^	15	1.23	2	1.69		7	1.41	1	0.84	
cirrhosis with portal hypertension	5	0.41	3	2.54		2	0.40	3	2.52	
steatosis (NASH/NAFLD)	22	1.80	0	0.00		8	1.61	1	0.84	
Smoking					0.251					0.422
no	1119	91.57	104	88.14		438	88.48	103	86.55	
in the past	60	4.91	10	8.47		34	6.87	12	10.08	
now	43	3.52	4	3.39		23	4.65	4	3.36	
Home medication										
ACEI					0.184					0.018
no	1051	85.87	96	81.36		382	76.86	79	66.39	
yes	173	14.13	22	18.64		115	23.14	40	33.61	
ARB:					0.377					0.356
no	1137	92.89	107	90.68		460	92.56	113	94.96	
yes	87	7.11	11	9.32		37	7.44	6	5.04	
Beta-blocker					0.029					0.026
no	985	80.47	85	72.03		306	61.57	60	50.42	
yes	239	19.53	33	27.97		191	38.43	59	49.58	
CCB					0.465					0.858
no	1114	91.01	105	88.98		388	78.07	92	77.31	
yes	110	8.99	13	11.02		109	21.93	27	22.69	
Alfa-blocker					0.903					0.082
no	1175	96.00	113	95.76		441	88.73	112	94.12	
yes	49	4.00	5	4.24		56	11.27	7	5.88	
Statin					0.522					0.062
no	1063	86.85	100	84.75		368	74.04	78	65.55	
yes	161	13.15	18	15.25		129	25.96	41	34.45	
Loop diuretic					0.14					0.12
no	1168	95.42	109	92.37		407	81.89	90	75.63	
yes	56	4.58	9	7.63		90	18.11	29	24.37	
Thiazides or thiazide-like diuretics					0.623					0.367
no	1145	93.55	109	92.37		452	90.95	105	88.24	
yes	79	6.45	9	7.63		45	9.05	14	11.76	
Course of hospitalization										
Pneumonia					<0.001					<0.001
no	628	51.31	29	24.58		225	45.27	34	28.57	
yes	596	48.69	89	75.42		272	54.73	85	71.43	
The most aggressive respiratory support					<0.001					<0.001
without oxygen therapy	574	46.90	11	9.32		214	43.23	28	23.73	
passive low-flow oxygen therapy	497	40.60	26	22.03		190	38.38	33	27.97	
passive high-flow oxygen therapy	71	5.80	16	13.56		37	7.47	7	5.93	
non-invasive ventilation (BiPAP/CPAP)	15	1.23	6	5.08		17	3.43	4	3.39	
respiratory therapy	67	5.47	59	50.00		37	7.47	46	38.98	
Transfer to ICU					<0.001					<0.001
no	1153	94.20	61	51.69		458	92.15	75	63.03	
yes	71	5.80	57	48.31		75	7.85	44	36.97	
Antibiotics					<0.001					<0.001
No	570	46.57	7	5.93		178	35.81	10	8.40	
yes	654	53.43	111	94.07		319	64.19	109	91.60	
Loop diuretics i.v.					0.14					0.12
No	1168	95.42	109	92.37		407	81.89	90	75.63	
yes	56	4.58	9	7.63		90	18.11	29	24.37	
Catecholamines					<0.001					<0.001
No	1169	95.51	56	47.46		446	89.74	71	59.66	
yes	55	4.49	62	52.54		51	10.26	48	40.34	

AKI—acute kidney injury; eGFR—estimated glomerular filtration rate (mL/min/1.73 m^2^); HFNC—High Flow Nasal Cannula; BiPAP/CPAP—Bilevel Positive Airway Pressure/Continuous Positive Airway Pressure; COPD-chronic obstructive pulmonary disease; NASH—non-alcoholic steatohepatitis; NAFLD—non-alcoholic fatty liver disease; ACEI—angiotensin-converting-enzyme inhibitor; ARB—angiotensin II receptor blocker; CCB—calcium channel blocker. ^1^ vaccination against SARS-CoV-2; ^2^ or cirrhosis without portal hypertension. Values were presented as number of observations (percent) * and mean ± SD.

**Table 10:** Laboratory values of patients with eGFR ≥ 60 mL/min/1.73 m^2^ at admission without and with AKI during hospitalization.

	Group A eGFR ≥ 60 Non-AKI (*n* = 1224)	Group A eGFR ≥ 60 AKI (*n* = 118)	*p*-Value
HGB, g/dL	13.50 (2.40)	12.55 (2.90)	≤0.0001
WBC, 10^3^/µL	6.90 (4.25)	8.97 (6.90)	≤0.0001
Lymphocytes, 10^3^/µL	1.02 (0.79)	0.88 (0.71)	0.0418
PLT, 10^3^/µL	215.00 (119.00)	224.00 (129.00)	0.573
Albumin, g/dL	3.30 (0.80)	2.90 (0.65)	≤0.0001
Total protein, g/dL	6.20 (6.70)	5.60 (1.10)	≤0.0001
CRP, mg/dL	40.71 (90.37)	95.29 (107.04)	≤0.0001
D-dimer, µg/mL	0.97 (1.68)	1.30 (4.37)	0.0133
Fibrinogen, g/L	4.65 (2.36)	5.09 (2.52)	0.125
IL-6, pg/mL	13.80 (30.23)	48.00 (90.60)	≤0.0001
Uric acid, mg/dL	4.80 (2.10)	4.55 (2.80)	0.847
Creatinine, mg/dL	0.82 (0.25)	0.82 (0.26)	0.491
Urea, mg/dL	31.00 (19.00)	38.00 (25.00)	≤0.0001
Potassium, mmol/L	4.00 (0.67)	4.10 (0.88)	0.0462
PCT, ng/mL	0.06 (0.10)	0.15 (0.44)	≤0.0001
Sodium, mmol/L	138.00 (5.00)	138.00 (6.00)	0.227
INR	1.09 (0.18)	1.18 (0.21)	≤0.0001
APTT	31.80 (8.00)	33.20 (11.70)	0.0438

eGFR: Estimated glomerular filtration rate (mL/min/1.73 m^2^); AKI—acute kidney injury; HGB: Hemoglobin; WBC: White blood cells; PLT: Platelets; CRP: C reactive protein; IL-6: Interleukin-6; PCT: Procalcitonin; INR: International normalized ratio; APTT: Activated partial thromboplastin time. Data are given as median and interquartile range.

**Table 11:** Comparison of outcomes between groups of COVID-19 patients divided according to eGFR level and appearance of AKI.

	Group A eGFR ≥ 60 (*n* = 1342)					Group B eGFR < 60 (*n* = 616)				
Variables	Non-AKI (*n* = 1224)		AKI (*n* = 118)			Non-AKI (*n* = 497)		AKI (*n* = 119)		
	*n*	*%*	*n*	*%*	*p*-Value	*n*	*%*	*n*	%	*p*-Value
In-hospital mortality					<0.001					<0.001
no	1159	94.69	44	37.29		388	78.07	47	39.5	
yes	65	5.31	74	62.71		109	21.93	72	60.5	
Death within 90 days of admission					<0.001					<0.001
no	1000	86.36	37	31.90		260	54.62	36	30.51	
yes	158	13.64	79	68.10		216	45.38	82	69.49	
Death within 180 days of admission					<0.001					<0.001
no	286	62.17	20	20.00		126	35.80	18	17.31	
yes	174	37.83	80	80.00		226	64.20	86	82.69	
End of hospitalization					<0.001					<0.001
discharge home	858	70.1	16	13.56		205	41.25	30	25.21	
emergency transfer to other centers	144	11.76	6	5.08		121	24.35	4	3.36	
transfer to other centers for rehabilitation	157	12.83	22	18.64		62	12.47	13	10.92	
death	65	5.31	74	62.71		109	21.93	72	60.50	
Number of hospitalization days ^1^	11 (2–15)		28 (13–32)		0.001	11 (2–15)		26 (12–34)		0.001

eGFR—estimated glomerular filtration rate (mL/min/1.73 m^2^). Values were presented as number of observations (percent). ^1^ median and interquartile range.

**Table 12:** Laboratory results of patients with eGFR < 60 mL/min/1.73 m^2^ at admission without and with AKI during hospitalization.

	Group B eGFR < 60 Non-AKI (*n* = 497)	Group B eGFR < 60 AKI (*n* = 119)	*p*-Value
HGB, g/dL	12.50 (3.50)	12.50 (3.60)	0.412
WBC, 10^3^/µL	7.83 (6.14)	8.75 (4.97)	0.022
Lymphocytes, 10^3^/µL	0.88 (0.77)	0.86 (0.80)	0.19
PLT, 10^3^/µL	201.00 (125.00)	196.00 (101.00)	0.641
Albumin, g/dL	3.00 (0.80)	2.90 (0.70)	0.135
Total protein, g/dL	6.00 (1.10)	5.50 (1.30)	0.0003
CRP, mg/dL	67.16 (121.87)	73.46 (121.15)	0.911
D-dimer, µg/mL	1.71 (2.70)	2.37 (5.20)	0.287
Fibrinogen, g/L	4.55 (2.99)	4.60 (2.34)	0.48
IL-6, pg/mL	23.00 (44.29)	30.85 (73.05)	0.172
Uric acid, mg/dL	6.90 (3.30)	7.30 (3.50)	0.516
Creatinine, mg/dL	1.62 (1.17)	1.83 (1.09)	0.022
Urea, mg/dL	74.00 (63.00)	80.00 (47.00)	0.171
Potassium, mmol/L	4.30 (0.91)	4.40 (1.04)	0.063
PCT, ng/mL	0.22 (0.64)	0.51 (1.03)	0.0001
Sodium, mmol/L	138.00 (6.00)	138.00 (6.00)	0.897
INR	1.17 (0.27)	1.18 (0.24)	0.855
APTT	33.00 (9.90)	33.40 (9.05)	0.835

eGFR: Estimated glomerular filtration rate (mL/min/1.73 m^2^); AKI—acute kidney injury; HGB: Hemoglobin; WBC: White blood cells; PLT: Platelets; CRP: C reactive protein; IL-6: Interleukin-6; PCT: Procalcitonin; INR: International normalized ratio; APTT: Activated partial thromboplastin time. Data are given as median and interquartile range.

## Data Availability

The datasets used and/or analyzed during the current study are available from the corresponding author upon reasonable request.
